# Transcriptional profiling of the LPS induced NF-κB response in macrophages

**DOI:** 10.1186/1471-2172-8-1

**Published:** 2007-01-12

**Authors:** Omar Sharif, Viacheslav N Bolshakov, Stephanie Raines, Peter Newham, Neil D Perkins

**Affiliations:** 1 Division of Gene Regulation and Expression, College of Life Sciences, University of Dundee, MSI/WTB/JBC Complex, Dow Street, Dundee, DD1 5EH Scotland, UK; 2 Post-Genomics and Molecular Interactions Centre, College of Life Sciences, University of Dundee, MSI/WTB/JBC Complex, Dow Street, Dundee, DD1 5EH Scotland, UK; 3 Target and Lead Generation, Respiratory and Inflammation Research Area, AstraZeneca, 8AF23, Alderley Park, Cheshire, SK10 4TG, UK; 4 Biocentre, University of Reading Whiteknights, PO Box 221, Reading, RG6 6AS, UK

## Abstract

**Background:**

Exposure of macrophages to bacterial products such as lipopolysaccharide (LPS) results in activation of the NF-κB transcription factor, which orchestrates a gene expression programme that underpins the macrophage-dependent immune response. These changes include the induction or repression of a wide range of genes that regulate inflammation, cell proliferation, migration and cell survival. This process is tightly regulated and loss of control is associated with conditions such as septic shock, inflammatory diseases and cancer. To study this response, it is important to have *in vitro *model systems that reflect the behaviour of cells *in vivo*. In addition, it is necessary to understand the natural differences that can occur between individuals. In this report, we have investigated and compared the LPS response in macrophage derived cell lines and peripheral blood mononuclear cell (PBMC) derived macrophages.

**Results:**

Gene expression profiles were determined following LPS treatment of THP-1 cells for 1 and 4 hours. LPS significantly induced or repressed 72 out of 465 genes selected as being known or putative NF-κB target genes, which exhibited 4 temporal patterns of expression. Results for 34 of these genes, including several genes not previously identified as LPS target genes, were validated using real time PCR. A high correlation between microarray and real time PCR data was found. Significantly, the LPS induced expression profile of THP-1 cells, as determined using real time PCR, was found to be very similar to that of human PBMC derived macrophages. Interestingly, some differences were observed in the LPS response between the two donor PBMC macrophage populations. Surprisingly, we found that the LPS response in U937 cells was dramatically different to both THP-1 and PBMC derived macrophages.

**Conclusion:**

This study revealed a dynamic and diverse transcriptional response to LPS in macrophages, involving both the induction and repression of gene expression in a time dependent manner. Moreover, we demonstrated that the LPS induced transcriptional response in the THP-1 cell line is very similar to primary PBMC derived macrophages. Therefore, THP-1 cells represent a good model system for studying the mechanisms of LPS and NF-κB dependent gene expression.

## Background

Primary human macrophages are sentinels of the immune system and following infection, circulating monocytes migrate from the peripheral blood to tissues where they differentiate into resident tissue macrophages [1]. Recognition of pathogen associated molecular patterns (PAMPS), including gram negative bacterial lipolysaccharide (LPS), results in activation of macrophages, leading to a plethora of biological responses required for shaping both the innate and adaptive arms of the immune response [2]. These effects are mediated through the release of chemokines and cytokines such as tumour necrosis factor α (TNF) and interleukin 1β (IL-1). However, excess release of these mediators can result in septic shock, multiple organ failure and acute respiratory distress syndrome [3]. Moreover, in mouse models of inflammation induced cancer, including an LPS induced model of lung metastasis, the release of cytokines by macrophages and host haematopoietic cells has been shown to stimulate tumour cell growth [4,5].

Many of the signalling events leading to cytokine synthesis and release following LPS exposure are now well established. Binding of LPS to toll-like receptor 4 (TLR4) activates two principal signalling pathways, distinguished by their dependence on the adaptor molecules myeloid differentiation factor 88 (MyD88) or TIR-domain-containing adaptor inducing IFN-β (TRIF) [6-10]. Significantly, both the Myd88 and TRIF pathways result in activation of the transcription factor, nuclear factor κB (NF-κB), a central regulator of the LPS, cytokine and stress responses in many cell types, including macrophages [8,9]. However, only the MyD88 independent, TRIF regulated, pathway is involved in the activation of IFN-β and interferon regulated genes such as CXCL10 via the transcription factor IRF-3. Differential signalling through MyD88 and TRIF results at least in part from the engagement of distinct downstream signalling proteins [10].

The mammalian NF-κB family consists of five members, RelA (p65), RelB, c-rel, p105/p50 (NFκB1) and p100/p52 (NFκB2) [11]. The NF-κB complex is composed of dimers formed from these subunits and in unstimulated cells is typically located in the cytoplasm bound to a member of the IκB family of proteins, α, β and ε. All IκB's contain ankyrin repeat, protein:protein interaction domains, a feature shared with the p100 and p105 precursor proteins which can also function in an IκB-like manner and retain their NF-κB partner subunits in the cytoplasm [11].

Many inflammatory stimuli, including exposure to LPS, TNF or IL-1, activate the canonical NF-κB signalling pathway. This pathway is characterised by the rapid phosphorylation and subsequent ubiquitin dependent degradation of IκBα by the 26S proteasome [11]. In some cell types, this can be followed by phosphorylation and degradation of IκBβ and ε, which contain analogous serine and lysine residues to those in IκBα required for phosphorylation induced ubiquitination. IκB phosphorylation by this pathway requires the IκB kinase (IKK) complex. This has three principal subunits, IKKα, β and the NF-κB essential modulator protein, NEMO (also known as IKKγ) [11]. Despite the high level of homology between IKKα and IKKβ, phosphorylation of IκB proteins during canonical pathway activation is performed almost exclusively by IKKβ [11]. IKKβ is also required for the phosphorylation and processing of the p105 subunit to p50 [12]. By contrast, IKKα is required for activation of the non-canonical pathway, which is induced by a subset of NF-κB inducers such as engagement of the CD40 and lymphotoxin-β receptors, B-cell activating factor, LPS, BAFF ligand and latent membrane protein (LMP)-1 of Epstein Barr virus (EBV) [11,13]. The non-canonical pathway is dependent on signalling through the NF-κB inducing kinase (NIK), which results in the activation of IKKα homodimers and the phosphorylation and 26S proteasome induced processing of the p100 NF-κB subunit to p52 [13].

The study of the NF-κB response in macrophages has often been limited to transformed cell lines. Such cell lines offer many advantages in terms of ease of use and can allow the biochemical dissection of many aspects of NF-κB signalling. However, their transformed nature might easily impact many aspects of NF-κB function, which is strongly influenced by oncogenes and tumour suppressors [12]. Indeed, differences in NF-κB signalling between transformed and primary cells have previously been noted [14]. Although primary human macrophages can be readily cultured *in vitro*, their numbers and lifespan are inherently limited. Furthermore, heterogeneity in macrophage responses between donors is well documented and arises as macrophages differentiate from their progenitors [15,16]. This can occur in part from genetic variations. For example, TLR4 mutations in humans can lead to decreased NF-κB DNA binding following LPS exposure [17]. Two immortalised human monocyte/macrophage cell lines commonly used by investigators to study the LPS response at various stages in the monocyte/macrophage differentiation process are U937 and THP-1 cells [18-21]. THP-1 cells have previously been shown to be a reasonably accurate model for native monocyte derived macrophages, as defined by an increase in adherence and surface expression of macrophage specific markers following treatment with phorbol-12-myristate-13 acetate (PMA) [22-24]. In this study, using a focussed microarray strategy and real-time PCR, we have characterised and compared the response to LPS in these cell lines and human primary, peripheral blood mononuclear cell (PBMC) derived macrophages. We find a surprisingly close correlation between THP-1 cells and PBMC derived macrophages, suggesting that for many aspects of the NF-κB response they provide a good model system for studying LPS induced changes in gene expression.

## Results

### Analysis of LPS-induced gene expression in the THP-1 macrophage cell line

**Figure 1:**
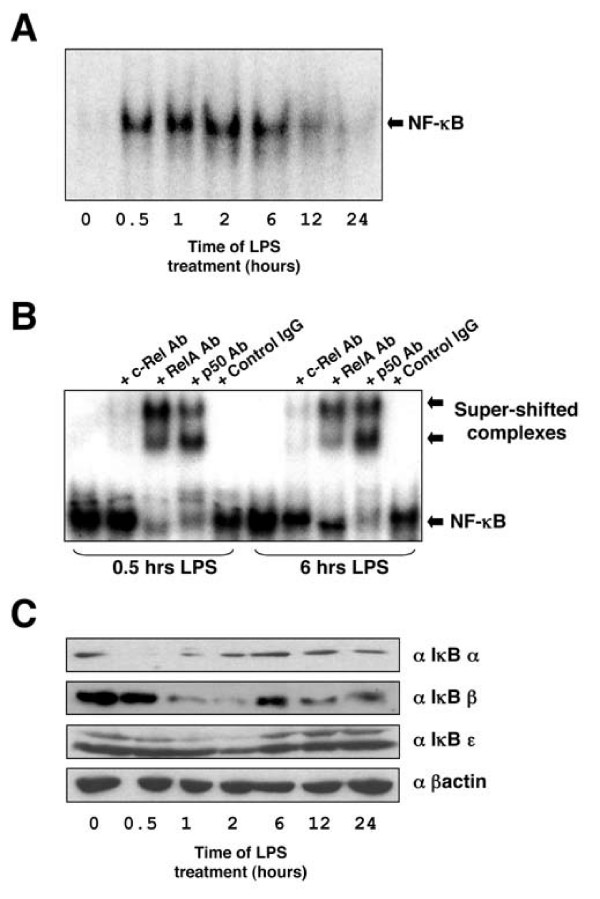
**Effect of LPS on NF-κB DNA binding and IκB protein degradation in THP-1 cells**. (A) THP-1 cells were treated with 2 μg/ml LPS for the times indicated. Nuclear extracts were prepared and assayed by EMSA with the HIV-1 κB probe. (B) Supershift analysis of the LPS induced NF-κB complex in THP-1 cells. (C) Effect of LPS on IκB degradation in THP-1 cells. THP-1 cells were treated 2 μg/ml LPS for the times indicated. Whole cell extracts were prepared and blotted using antibodies specific for IκB-α, IκB-β and IκB-ε.

Before analysis of LPS-induced changes in gene expression in the THP-1 macrophage cell line could begin, it was first necessary to characterise the NF-κB response in these cells. Electrophoretic mobility shift analysis (EMSA) demonstrated that LPS rapidly induces NF-κB DNA-binding. In these and all subsequent experiments we did not differentiate the THP-1 cells with PMA, as it is both an activator of NF-κB [25] and has been shown to enhance levels of gene expression in response to LPS [26]. This activity persists at a high level for a number of hours before beginning to decrease 12 hours after stimulation and being lost entirely after 24 hours (Figure 1A). Supershift analysis demonstrated that at both early and late time points this NF-κB complex consisted primarily of the p50 and RelA subunits, although some c-Rel could also be detected (Figure 1B). The presence of p52 and RelB was not analysed and cannot be ruled out. To further clarify this response, IκB protein levels were investigated. Rapid degradation and resynthesis of IκBα was observed followed by a slower loss of IκBβ and ε, consistent with previous reports (Figure 1C) [27,28]. It should be noted that the upper band in the IκBε blot corresponds to a phosphorylated form and has previously been detected using this antibody [28]. Consistent with activation of the canonical NF-κB pathway, an IKKβ inhibitor [29] blocked both IκB phosphorylation at serine 32/36, degradation and induction of NF-κB DNA-binding (data not shown).

Using this information, RNA was extracted from two separate batches of THP-1 cells, to create two biological replicates, either stimulated with LPS for 1 or 4 hours or left untreated. The two biological replicates were used to ensure that any results were not biased by day-to-day variation in RNA extraction [30,31]. The RNA was post-labelled, ensuring both optimal yield of cDNA, random attachment and equal labelling of samples [30,31].

To analyse LPS induced gene expression changes, we used a highly focussed microarray containing PCR amplified DNA fragments from genes with either predicted or known NF-κB binding sites in their upstream sequences [See Additional file 1]. These were identified either from a previous review of NF-κB regulated genes [25] or from our own analysis of the literature (Pubmed links for each gene describing their association with NF-κB can be found in the second sheet of Additional file 1). Also included were genes without known κB elements but which belonged to the same general gene families, such as chemokine genes and their receptors. Such focussed arrays have previously been used as an economical approach for detailed pathway analysis of a carefully selected subset of genes [18]. Following labelling, samples were competitively hybridised to the arrays. Following hybridisation, normalisation and data analysis, only genes with significant (p < 0.05), as determined using the 1 sample T test, and with average ratios of more than log_2 _1 or less than -1, were considered as more than two fold up or down-regulated respectively [see Additional file 2].

**Figure 3:**
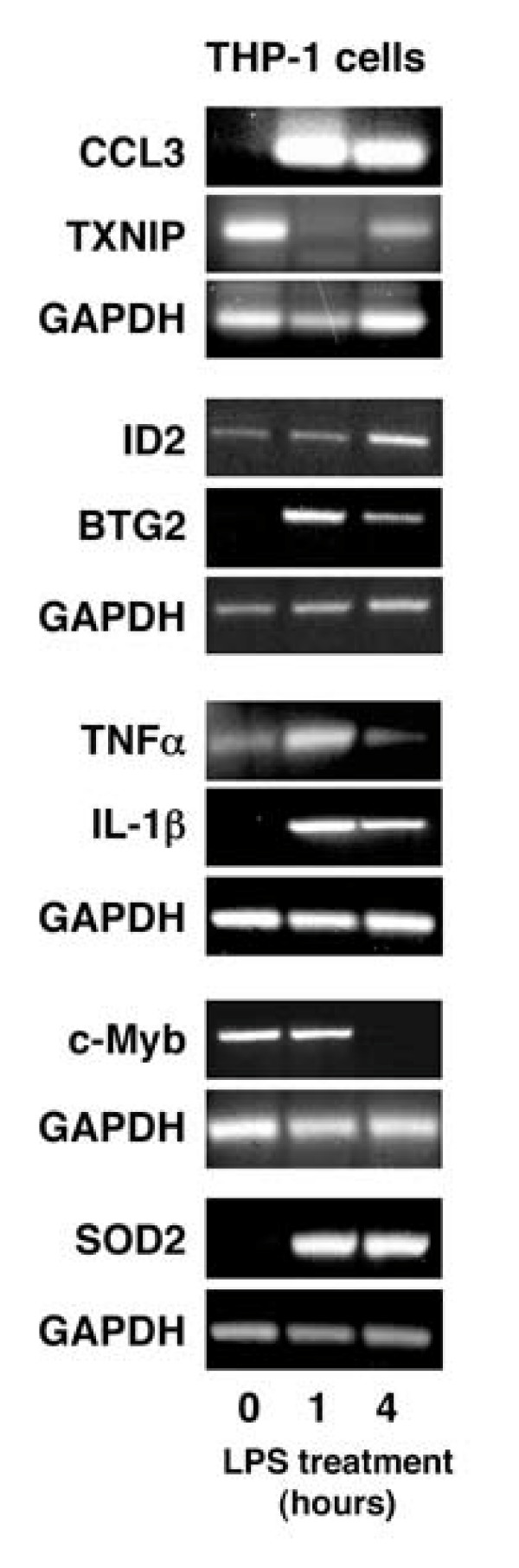
**Validation of microarray results using semi-quantitative RT-PCR**. THP-1 cells were treated with 2 μg/ml LPS for 1 hour or 4 hours and RNA extracted. This was subjected to semi-quantitative RT-PCR using primers specific for each of the genes shown. Data is representative of 3 independent experiments. Experiments were performed on separate days and GAPDH controls for each distinct experiment are shown.

Using these criteria, 72 out of the 465 genes on the array were found to significantly respond to LPS treatment in THP-1 cells. These can be grouped into four temporal patterns of expression (Figure 3). The most common patterns were genes whose expression was unaffected after 1 hour of LPS treatment but showed either a decrease (25 out of 72 genes) or an increase (19 out of 72 genes) 4 hours post-treatment. The next most common pattern of expression consisted of genes whose expression was induced following 1 hour of LPS treatment and where, although frequently still higher than in untreated cells, expression decreased 4 hours post-treatment (18 out of 72 genes). 8 out of 72 genes exhibited a profile of being induced following 1 hour of LPS treatment with expression rising further after 4 hours stimulation with LPS. The least common pattern of expression were genes whose expression was repressed following 1 hour LPS treatment but which showed an increase in expression, relative to untreated cells, after 4 hours (Fig 3). These 4 temporal patterns of expression are consistent with previously reported observations in LPS treated, PMA differentiated THP-1 cells [18,19].

### Most genes responsive to LPS in THP-1 cells contain known or putative κB sites

The genes affected by LPS treatment can be further divided into two different categories. Those that have been previously characterised as NF-κB target genes or which contain putative κB sites in their upstream regulatory regions, as determined by transcription factor prediction analysis (Table 1, 47 out of the 72 genes), and those that do not contain putative κB sites (Table 2, 25 out of the 72 genes) [See also Additional file 1]. It cannot be ruled out that there are genes in this latter category that contain cryptic κB elements or where the NF-κB binding site is contained in an intronic or remote enhancer motif. The number of genes in the first category correlates with the observation that, in B-cells, NF-κB is a master regulator of LPS responses [32].

IRF-3 is a transcription factor that is induced in a TRIF-dependent but MyD88 independent manner and is associated with the induction of later LPS inducible genes [9]. Interestingly, most of the genes listed in Table 2 are only responsive to LPS at 4 hours (Figure 3). We therefore searched for potential IRF-3 regulatory sites in these genes by transcription factor prediction analysis and found that many of these contain putative IRF-3 sites in their upstream sequences, consistent with a potential role of IRF-3 in their later LPS responsiveness (Table 2, indicated by #, see also Additional File 1 for positions of sites). Interestingly, RelA and IRF-3 interact following LPS treatment and promote IRF-3 mediated transcription [33], suggesting that these genes, albeit indirectly, might still be regulated by NF-κB.

### Validation of microarray results

**Figure 4:**
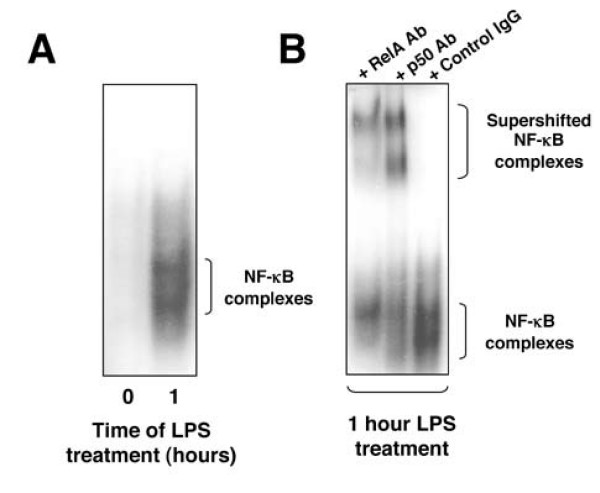
**Effect of LPS on NF-κB DNA binding in PBMC derived macrophages**. (A) PBMC derived macrophages were isolated and were treated with 2 μg/ml LPS. Nuclear extracts were prepared and assayed using EMSA with the HIV-1 probe (A). (B) Supershift analysis of the LPS induced NF-κB complex in PBMC derived macrophages.

Microarray data is subject to considerable variability, particularly with spotted microarrays from different laboratories or among different platforms [34,35]. The four temporal patterns of gene expression observed in this study (Figure 3) have previously been observed [18,19]. However, among the individual genes some discrepancies with previous reports are apparent, although these may reflect the use of PMA differentiated THP-1 cells, for example [18]. Therefore, it was necessary to validate the microarray results using another experimental approach. Initially this was performed using semi-quantitative PCR on a subset of the genes studied. This approach confirmed both the four temporal patterns of expression and that expression of CCL3, TNF-α and IL-1β, which differed from that reported previously [18] was correct (Figure 4).

To obtain an accurate picture of differential gene regulation and, importantly, to screen a larger number of genes simultaneously, we next used a Taqman microfluidics, real-time PCR based strategy. 34 of the genes identified in the initial microarray screen were selected for inclusion on the microfluidics cards for further validation. While similar patterns of gene expression were generally identified between the microarray and real time PCR analysis, some differences were observed (Table 3, see also Additional File 2). In particular, because of the increased sensitivity found with Taqman a greater fold change in gene expression is observed relative to the results obtained using microarray. This is consistent with the previously reported fold change bias in microarray experiments [36] and can result in some genes being scored as no change in microarray but where a real difference in expression can be observed using real time PCR. However, there were only 12 genes whose expression profile differed between the two techniques. Out of these, 9 resulted from the reason just described, while there were only 3 (JARID1B, HLADRα and SMYD5) that were found to be false positives at both LPS time points (Table 3). Importantly, this analysis confirmed the LPS responsiveness of the genes CCL3L1, TXNIP, PDCD4, C-MYB, TOP2α, BUB1β and ID2. These genes have not, to the best of our knowledge, been previously identified as LPS responsive genes.

### Comparison of gene expression in THP-1 cells to PBMC derived macrophages

In terms of expression of cell surface markers, PMA differentiated THP-1 cells have previously been shown to be a reasonably accurate model for native, monocyte derived, macrophages [22]. Therefore, we next examined, at the gene expression level, how the LPS responses of THP-1 cells and primary PBMC human macrophages compared. First, EMSA confirmed that the PBMC macrophages we isolated had low basal levels of NF-κB, which could be strongly induced upon LPS treatment (Figure 4). Supershift analysis further demonstrated that this NF-κB complex consisted primarily of RelA and p50 (Figure 4). Although other complexes are visible, these were not characterised further. Next, using Taqman microfluidics real-time PCR, we analysed the expression of the 34 genes investigated in THP-1 cells above. Broadly speaking there was good agreement between the results obtained in THP-1 cells and two separate donors of PBMC derived macrophages, although in a number of cases, CCL3, CCL4, CXCL8, IL-1β, IL-1RN, COX-2, SOD2, BTG2 and IGFBP3, while the genes were similarly induced or repressed there were significant differences in relative fold induction (Table 4). In addition, some genes, CCL22, CXCR4, IL-1β, c-Myb, BTG2 and JARID1B, showed evidence of significant donor to donor variability, although in many cases the fold changes seen with these genes were low to begin with. Only 4 genes, CCL22, PMAIP1, HLADRα and SMYD5 showed significantly contrasting results between THP-1 cells and both donor samples, although in these latter two cases the fold changes were again small. Significantly, these results confirmed that the novel LPS responsive genes CCL3L1, PDCD4, TOP2α, BUB1β and ID2 identified in THP-1 cells, exhibit an almost identical expression profile to primary human macrophages.

### Comparison of gene expression in U937 cells to PBMC derived macrophages

The U937 cell line, which like THP-1 cells have previously been shown to express TLR4, irrespective of differentiation status [21], is also a frequently used cell line to study the LPS response [22-24]. We therefore also determined the expression profile of the same set of 34 genes using real time PCR in this cell type. Surprisingly, we found that in contrast to THP-1 cells and PBMC derived macrophages, LPS mediated gene expression in U937 cells is very different. Most of the genes tested were not responsive to LPS or were induced at very low levels (Table 4). Only 7 genes, CXCL2, TNF-α, cIAP2, SOD2, TNFAIP3, IκB-α and ICAM1, showed an identical expression profile between THP-1 cells, U937 cells and PBMC derived macrophages from both donors (Table 4). None of the novel LPS responsive genes in THP-1 cells and PBMC derived macrophages described above are expressed in U937 cells following LPS treatment.

## Discussion

In this report we have characterised the LPS response in macrophages and macrophage cell lines. Strikingly, we observed that although THP1 cells are transformed and immortalised, their LPS induced gene expression signature remains very similar to primary macrophages. Interestingly, there was also some heterogeneity between the two different PBMC donor samples. This donor-to-donor variation is not really surprising since macrophage heterogeneity occurs through a developmental mechanism as they differentiate and is likely to be important for the generation of diversity in the immune response [15]. For example, different populations of macrophages have been shown to exhibit different methylation patterns and this has been implicated in modulating the LPS response [37,38]. Moreover, even different sub-clones of RAW264 macrophages have been reported to respond differently to LPS by expressing different genes [16]. Therefore, although NF-κB is a master regulator of the LPS response [32], other factors such as the chromatin structure of gene promoters will influence whether transcription initiation occurs. Indeed, following LPS treatment, a subset of genes exist which require chromatin remodelling events to allow access of NF-κB and the RNA polymerase II machinery to their promoters at later time points [39,40].

By contrast with the results from THP1 cells and PBMCs, the U937 cell line showed dramatic differences in the gene expression changes induced in response to LPS treatment. This may reflect differences in the differentiation status of these cells or epigenetic differences in the chromatin structure at the promoter levels of these genes. For example, differentiation has been shown to increase expression of CD14, an LPS receptor [23,41,42] but in this study both the THP-1 and U937 cells were not treated with differentiating agents (such as PMA) prior to LPS stimulation. Therefore, these differences may reflect differing levels of CD14 cell surface expression. Alternatively, these results might reflect clonal variations between U937 cells used in different laboratories.

In other systems, the LPS response has been shown to depend almost entirely on the initial activation of the NF-κB/IKK pathway [32]. Indeed, the use of a highly selective IKK inhibitor [29] confirmed that the expression of almost all the genes validated by real-time PCR are NF-κB/IKK-dependent (data not shown). However, it cannot be concluded that all these genes are direct NF-κB targets. Although many of these genes contain known or putative NF-κB sites in their promoters, many others do not (Tables 1 &2) and may therefore be targets of transcription factors, such as AP-1 or IRF-3, activated as a secondary consequence of the initial wave of NF-κB induction [9,32]. A recent study using a ChIP-on-chip approach identified hundreds of direct NF-κB targets following LPS stimulation of U937 cells [24]. Interestingly, 157 genes were bound by NF-κB subunits prior to LPS treatment, and after 1 hour of LPS stimulation this increased to 326 genes. Importantly, in the unstimulated cells, it was the p50 NF-κB subunit that bound these promoters and RelA recruitment was only observed following LPS treatment [24]. Among the promoters demonstrated to recruit RelA were those for the CCL3, CCL4, ICAM1, IL-1β, CXCL8 and TNF-α genes [24], all of which we also found to upregulated following 1 hour of LPS treatment. However, in contrast to this study, we found no upregulation of GADD45β or PMAIP1 expression in U937 cells, although these genes were demonstrated to recruit RelA following LPS treatment [24]. This may be related to the differences of experimental conditions, such as serotype of LPS. It should also be noted that some commonly used commercial preparations of LPS can contain other, contaminating, bacterial products such as peptidoglycans and RNA [43]. These can lead to signalling through TLR2 in addition to TLR4 [43] and we cannot rule out that our results might reflect the activation of such additional signalling pathways.

In addition to inflammatory mediators, LPS induced genes with a wide variety of different functions. These included genes associated with cell stress (SOD2, TXNIP), apoptosis (GADD45β, cIAP2, TNFAIP3), the cell cycle and differentiation (BTG2, ID2). In addition, the products of many genes have the potential to be involved in feedback loops. For example, inflammatory molecules such as TNF-α, IL-1β and CCL19 have been shown to promote NF-κB signalling [44-46]. Excessive release of cytokines is associated with the adverse clinical consequences of infection such as sepsis and shock [3]. Therefore, it was of interest to also find a plethora of LPS responsive genes that can limit feedback signalling. IL-1RN is a protein that binds to the IL-1R, inhibiting binding of IL-1β and thus neutralising the biological activity of this cytokine [47]. The anti-apoptotic gene TNFAIP3 is involved in the termination of TLR-induced NF-κB activity and pro-inflammatory gene expression, in macrophages, and protects against endotoxin shock [48]. The pro-apoptotic gene PBP can also interact with upstream components of the NF-κB signalling pathway, including NIK, MEKK1 and TAK1 [11], and therefore inhibits NF-κB activation induced by TNF-α and IL-1β [49]. Furthermore, similar to PBP, the pro-apoptotic gene TRIP also inhibits NF-κB signalling [50].

It is also of interest, that many genes associated with an anti-apoptotic response such as TNFAIP3, GADD45β, cIAP2 [48,51,52] were induced by LPS whereas those genes that are associated with being pro-apoptotic, such as PBP, PDCD4 [53,54], were repressed. This also correlates with the later co-expression of CCL19 and its receptor CCR-7, which has been shown to promote NF-κB signalling and an anti-apoptotic response [46]. Indeed, delayed apoptosis observed in patients with bacterial sepsis has been reported to correlate with expression of the cytokine PBEF [55], which we find to be expressed in response to LPS. Although these results are consistent with the widely reported anti-apoptotic effects of NF-κB [56], it is interesting to note that some well known anti-apoptotic target genes, such as Bcl-xL, were not significantly induced by LPS in THP-1 cells [see Additional file 2].

## Conclusion

Although this study examined only a limited set of genes a number of discoveries were made and some significant conclusions can be drawn. Firstly, the temporal nature of LPS induced gene expression allowed four distinct patterns of gene expression to be defined. Additional time-points may reveal further subtleties but it is clear that the changes in gene expression following LPS stimulation are dynamic and feature both the time-specific repression as well as activation of gene expression. However, it should be noted that gene expression patterns do not necessarily correlate with protein levels [57]. This analysis also allowed us to conclude that THP-1 cells provide a much better model system for evaluating the LPS response in macrophages than U937 cells: relatively few differences were observed between THP-1 cells and primary macrophages. These included a number of novel LPS-responsive genes identified in this study, CCL3L1, TXNIP, PDCD4, C-MYB, TOP2α, BUB1β and ID2,. Inhibitors of the NF-κB/IKK pathway have many potential uses, including the treatment of inflammatory diseases, such as rheumatoid arthritis and cancer. In many of these diseases, cytokine and chemokine production by macrophages have been shown to play a key pathological role [4,5]. While analysis of primary macrophages in culture will always provide more physiologically relevant information than cultured cell lines, it is also the case that primary cells are hard to culture for long periods and in the quantities required to permit biochemical analysis. The confirmation here that, in many respects, THP-1 cells are a valid model system for studying the LPS response in macrophages, will therefore give confidence that the conclusions drawn from such studies can be applied *in vivo*.

## Methods

### Cell lines

THP-1 and U937 cells were cultured in RPMI 1640 medium supplemented with 10% heat inactivated foetal calf serum (FCS), 1% Penicillin and Streptomycin and 5 mM Glutamine (PSG) at 37°C, 5% CO_2_. For cell treatment, 1 × 10^7 ^cells were treated with 2 μg/ml LPS 0111:B4 (Sigma, cat no L4391, purified by phenol extraction and gel filtration) for the appropriate times.

### Isolation and culture of PBMC derived macrophages

Buffy coat preparations used were all transported at room temperature and were 1 day old (to allow time for testing for HIV and HepC). 80–100 ml of buffy coat (Ninewells Blood Transfusion Service, Dundee) was diluted to a final volume of 250 ml, with room temperature PBS supplemented with 10 mM glucose and 5 mM EDTA. 30 ml was layered onto 15 ml of Ficoll and peripheral blood mononuclear cells (PBMCs) were isolated by density centrigugation according to the manufacturers instructions (Amersham). Monocytes were isolated from PBMCs by adherence, as described previously [58]. Briefly, 1.5 × 10^8 ^PBMCs, resuspended in 10 mls of 37°C RPMI supplemented with 10% heat inactivated FCS and 1% PSG, were plated in tissue culture dishes and placed in a 37°C incubator for 1 hour to allow adherence to take place. Non-adherent cells were removed by gentle re-suspension and adherent cells were washed quickly, but gently, in 37°C media, until no free-floating cells were observed using light microscopy. Washing was repeated a total of 4 times, followed by a 20 minute incubation at 37°C to prevent monocyte loss during the washing steps. The cells were then washed a further 4 times, until a homogenous population of monocytes was detected by light microscopy. Following overnight culture, media was replaced by media supplemented with 10 ng/ml macrophage colony stimulating factor (M-CSF, Sigma). Cells were cultured for 7 days with fresh addition of media/M-CSF every couple of days. Isolation and culture of PBMC derived macrophages from both donors was conducted at the same time.

### Western Blot analysis

After treatment, THP-1 cells were washed once with cold PBS, solubilized by resuspending the pellet in ice cold whole cell extract buffer (20 mM Hepes pH 7.6, 400 mM NaCl, 1 mM EDTA, 5 mM NaF, 500 μM Na_3_VO_4_, 25% glycerol, 0.1% NP-40, 1 mM PMSF, 1 mM DTT, 0.1 mg/ml aprotonin) and passed through a 25G needle 10 times, as described [59]. Lysates were centrifuged at 14,000 rpm for 15 minutes. 25 μg of supernatant was separated by electrophoresis on a 10% SDS polyacrylamide gel and transferred to polyvinylidene difluoride (PVDF) membranes. Antibodies specific for IκB-α, IκB-β, IκB-ε were kindly donated by Dr Nancy Rice (NCI, USA) and used at dilutions of 1:1000. Anti-Sera directed against β-actin were obtained from Cell Signalling and used at 1:2000 dilution. Immunoreactive proteins were detected by enhanced chemiluminescent protocol (Amersham).

### Electrophoretic Mobility Shift Assay (EMSA)

After treatment, nuclear extracts were prepared by washing cells once with cold PBS, resuspending the cell pellet in ice cold low salt buffer (10 mM Hepes pH 7.9, 1.5 mM MgCl_2_, 10 mM KCl, 1 mM DTT, 1 mM PMSF, 1 μg/ml leupepetin, 1 μg/ml aprotonin, 1 μg/ml pepstatin, 1 μg/ml E64) and passed through a 25G needle 10 times, as described [59]. Lysates were centrifuged at 14,000 rpm for 10 minutes and the cytoplasmic fraction was removed. The nuclear pellets were resuspended in high salt buffer (20 mM Hepes pH 7.9, 1.5 mM MgCl_2_, 420 mM NaCl, 1 mM DTT, 1 mM PMSF, 1 μg/ml leupepetin, 1 μg/ml aprotonin, 1 μg/ml pepstatin, 1 μg/ml E64, 25% glycerol), rocked at 4°C for 15 minutes and centrifuged at 14,000 rpm, for 15 minutes. Supernatants were removed and stored at -80°C prior to running the EMSA. 10 μg of nuclear extract was used in each EMSA reaction using a synthetic double stranded ^32^P end labelled probe containing the NF-κB binding site of the HIV-1 LTR (5'GATCCGCTGGGGACTTTCCAGCGC3'). For the binding assay, nuclear extract was added to 20 mM Hepes pH7.9, 125 mM NaCl, 1 mM DTT, 1 μg of poly(dI-dC) and 0.1 ng probe and incubated at room temperature for 15 minutes. For supershift assays 0.5 μl of anti-sera against RelA, p50 or c-Rel or control IgG antibody were added before addition of the probe. Samples were separated on a pre-run nondenaturing polyacrylamide gel in TGE buffer (25 mM Tris-HCl pH8.0, 190 mM glycine, 1 mM EDTA) for 4 hours at 150V. The gel was dried and NF-κB DNA binding was visualised by autoradiography.

### Microarray printing, labelling and hybridisation

A total of 465 PCR-amplified cDNA clone fragments were obtained from AstraZeneca, dissolved in 3xSSC printing buffer and printed in triplicate onto Corning GAPS II Coated Slides (Corning Life Sciences, Corning, NY, USA). Printed arrays were processed and stored according to the slide manufacturer's instructions. Total RNA was labelled with CyScribe Post Labelling Kit (Amersham) as recommended by the manufacturer's protocol, except that synthesized cDNA was purified by ethanol precipitation and the labelled target was purified with QIAquick PCR Purification Kit (QIAGEN). Microarray pre-hybridization, hybridization and washes were performed according to protocols and conditions previously described [60], except that the hybridization buffer contained 50% formamide, 5% dextrane sulfate, 5× Denhardt's solution, 3× SSC, 1% SDS, and all washes were done at room temperature. Each experiment was performed in two biological replicates, with two technical (dye-swap) replicates for each.

### Microarray data acquisition and analysis

Microarrays were scanned using an ArrayWoRx^*e *^scanner and images were analysed with ArrayWoRx Software (Applied Precision LLC). Raw spot intensity data were imported in locally installed BASE [61] and pre-processed by local background subtraction, quality filtering (SNR > 3), spot averaging and calculating raw experiment/control ratios. Tables with ratios for spots with two or more experimental replicates were imported into and analysed with locally installed TIGR Multiexperimental Viewer (TMeV) software package [62]. First, data were adjusted by log_2_-transformation and global median normalization. Then, differentially expressed genes between experimental and control conditions were determined with one-sample t-test (P < 0.05, > 2-fold change). Selected genes were annotated using public databases and additional literature search. The microarray data have been deposited in ArrayExpress (accession no: E-MEXP-868) [63].

### Real time quantitative PCR

After treatment, total RNA was extracted from THP-1 cells using the RNeasy Mini Kit (QIAGEN) according to the manufacturers instructions, which included a DNase step. Purified RNA was quantified and assayed for degradation using an agilent bioanalyzer (Agilent) according to the manufacturers instructions. 125 ng of total RNA was used to determine gene expression levels by Taqman analysis using the ABI PRISM 7900HT sequence detection system. For analysis, a threshold was set for the change in fluorescence at a point in the linear PCR amplification phase. The level of RNA for treated cells compared to untreated cells and normalised to GAPDH was calculated using the SDS 2.1 software (Applied Biosystems) which relies on the comparative Ct method of quantification ()[64]. Genes that exhibited a mean fold change between 2 and -2 were considered to be unaffected. Human primers and probes specific for each of the genes and housekeepers were obtained as assay on demands on microfludic cards [65]. Cycling conditions were set at 35 cycles of 50°C for 30 min, 94.5°C for 15 min, 97°C for 30 seconds and 59.7°C for 1 minute.

### Semi-quantitative PCR

Semi-quantitative RT-PCR was carried out using the AccessQuick™ RT-PCR system according to the manufacturers instructions (Promega). Briefly, 10–100 ng of RNA was added to a tube containing 1 × reaction buffer, 50 pmol primers and 5U AMV reverse transcriptase in a final volume of 50 μl. Cycling conditions were 48°C for 45 minutes, 94°C for 2 minutes, then 40 cycles of 94°C for 1 minute, 60°C for 1 minute, 68°C for 2 minutes and 1 cycle of 68°C for 5 minutes. Human gene specific primer sequences used were; SOD2 forward (5'CAGATCATGCAGCTGCACCAC3'), reverse (5'GTAGTAAGCGTGCTCCCACAC3'); IL-1β forward (5'GCCATGGACAAGCTGAGGAAG3'), reverse (5'GTGCTGATGTACCAGTTGGG3'); TNF-α forward (5'GCGTGGAGCTGAGAGATAACC3'), reverse (5' GATCCCAAAGTAGACCTGCCC3'); TXNIP forward (5'CTATCCTGGGCTGCAACATCC3'), reverse (5' GTTGAGGATGCAGGGATCCAC3'); CCL3 forward (5' CTTGCTGTCCTCCTCTGCAC3'), reverse (5'TCACTGGGGTCAGCACAGAC3'); C-MYB forward (5' ACTTCCACCCCCCTCATTGG3'), reverse (5'GCTCCTCCATCTTTCCACAGG3'); ID2 forward (5'GAAAGCCTTCAGTCCCGTGAG3'), reverse (5' TCCGTGTTGAGGGTGGTCAG3'); BTG2 forward (5'GGAAGGGAACCGACATGCTC3'), reverse (5'CTAGCTGGAGACTGCCATCAC3') and GAPDH forward (5'GGTCGTATTGGGCGCCTGGTCACC3'), reverse (5'CACACCCATGACGAACATGGGGGC3')

### Identification of NF-κB and IRF-3 promoter binding sites

NF-κB binding sites were identified as previously described [66]. 2 Kb of genomic sequence upstream from the initiator ATG codon of each gene on the array was determined [see Additional File 1]. This sequence was analysed with Gene Runner software (Hastings Software, Hastings on Hudson, NY, USA) to search for the κB motif 5'GGGRNNYYCC3' [66]. The same sequence was used to search for the IRF-3 motif 5'GAAANNGAAANN3' [67].

### Data deposition

The microarray data have been deposited in ArrayExpress (accession no: E-MEXP-868) [63]

## Authors' contributions

OS performed EMSAs, western blots, RNA extractions and hybridisation of the arrays, semi-quantitative and real time PCR, promoter analysis for NF-κB and IRF-3 binding sites and participated in experimental design and writing of manuscript. VNB was performed the printing and processing of microarrays, microarray image acquisition and analysis, microarray data processing and analysis and also articipated in experimental design. SR provided assistance with Taqman real-time PCR data analysis. PN provided the cDNAs for the microarray and reagents and equipment for real-time PCR analysis and also articipated in experimental design. NDP is the principal grant holder and participated in experimental design and writing of manuscript. All authors read and approved the final manuscript.

## Supplementary Material

Additional File 1 Genes used for microarray analysis. Genes with either predicted or known NF-κB binding sites in their upstream sequences were used for the microarray in this study. File contains gene names, accession numbers, location of putative κB and IRF sites and Pubmed links to reports describing regulation by NF-κB (where available). Click here for file

Additional File 2 Microarray data. The hybridization (expression) data for each gene included on the microarray. Click here for file

## Figures and Tables

**Figure 2:**
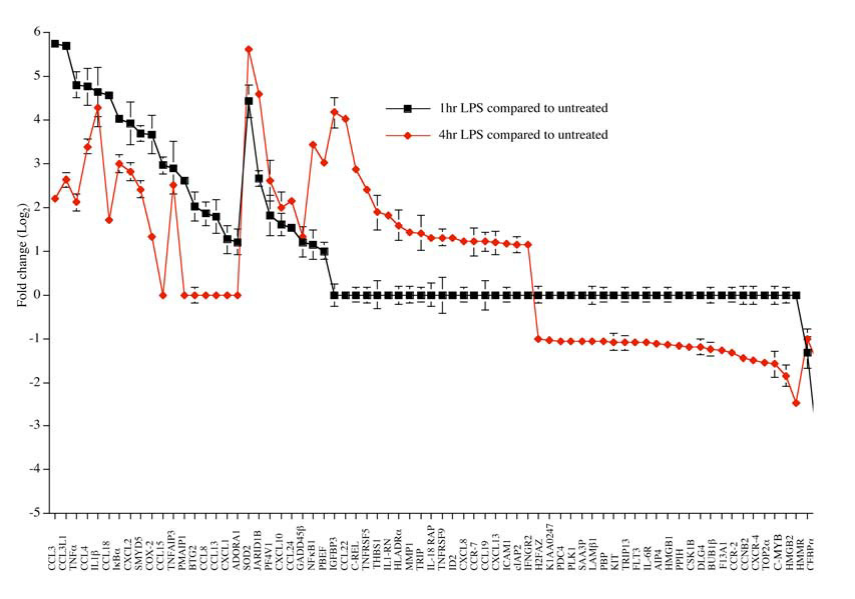
**Temporal patterns of gene expression in LPS stimulated THP-1 cells**. Only genes with an average log_2 _ratio more than 1 or less then -1 at either 1 hour (Black line) or 4 hour (Red line) LPS time point relative to the control (P < 0.05, one-sample t test) are shown. If genes had the ratio more than -1 or less then 1 for a particular time point, they were considered unchanged and their ratios were set to 0. Error bars represent the SEM. Data is representative of 8 microarray experiments, 2 biological replicates and 2 technical replicates for each time point, and actual ratios for all genes in all replicates are presented in Additional file 2

**Table 1:** Genes responsive to LPS that are known NF-κB targets or that have putative sites κB sites

Accession no	Symbol	Gene Name	Reference
**Chemokines and receptors**			
[genebank:NM_002983]	CCL3	Chemokine, CC motif, Ligand 3 π	[25]
[genebank:NM_002984]	CCL4	Chemokine, CC motif, ligand 4 π	[25]
[genebank:NM_005408]	CCL13	Chemokine, CC motif, ligand 13	[68]
[genebank:NM_032964]	CCL15	Chemokine, CC motif, ligand 15	[69]
[genebank:NM_006274]	CCL19	Chemokine, CC motif, ligand 19	-1748*
[genebank:NM_002990]	CCL22	Chemokine, CC motif, ligand 22 π	[70]
[genebank:NM_001511]	CXCL1	Chemokine CC motif, Ligand 1 π	[25]
[genebank:NM_002089]	CXCL2	Chemokine, CXC motif, ligand 2 π	[25]
[genebank:NM_000584]	CXCL8	Chemokine, CXC motif, ligand 8 π	[25]
[genebank:NM_001565]	CXCL10	Chemokine, CXC motif, ligand 10	[25]
[genebank:NM_005408]	CXCL13	Chemokine, CXC motif, ligand 13	-1593*
[genebank:U28934]	CCR2	Chemokine, CC motif, receptor 2	-1543*
[genebank:NM_003467]	CXCR4	Chemokine, CXC motif, receptor 4 π	[71]
**Cytokines and Receptors**			
[genebank:NM_000594]	TNF-α	Tumour Necrosis Factor α π	[25]
[genebank:NM_000576]	IL1-β	Interleukin 1 B π	[25]
[genebank:NM_000577]	IL1-RN	Interleukin 1 receptor antagonist π	[25]
[genebank:NM_005746]	PBEF	Pre B cell colony stimulating factor 1 π	-533*, -479*
[genebank:NM_001250]	TNFRSF5	TNF receptor superfamily, member 5	[72]
[genebank:NM_001561]	TNFRSF9	TNF receptor superfamily member 9	[73]
[genebank:NM_005534]	IFNGR2	Interferon, gamma receptor 2	-317, -220*, -144, -134*
**Stress**			
[genebank:NM_000636]	SOD2	Superoxide dismutase 2 πp	[25]
[genebank:NM_000963]	COX2	Cycloxygenase 2 π	[25]
[genebank:NM_006427]	TXNIP	Thioredoxin interacting protein π	-618
**Apoptosis**			
[genebank:NM_015675]	GADD45 β	Growth arrest and DNA damage inducible gene GADD45, B π	[74]
[genebank:NM_005879]	TRIP	Traf interacting protein	-1736
[genebank:NM_006290]	TNFAIP3	Tumour Necrosis factor α induced protein 3 π	[25]
[genebank:NM_001165]	cIAP2	Cellular inhibitor of apoptosis 2 π	[75]
**Transcription**			
[genebank:NM_020529]	IκB-α	Inhibitor of κlight chain gene enhancer in B cells; α π	[25]
[genebank:NM_003998]	NFκB1	Nuclear factor of κB, subunit 1 π	[25]
[genebank:NM_002908]	C-REL	V-rel avain reticuloendotheliosis viral oncogene homolog; rel π	[25]
[genebank:NM_005375]	C-MYB	V-MYB avian myeloblastosis viral oncogene homolog π	[25]
[genebank:NM_004364]	CEBPα	CAAT/enhancer binding protein α	[25]
[genebank:U96131]	TRIP13	Thyroid hormone interactor 13	-1611, -1601*, -1600* -1464*
[genebank:NM_002129]	HMGB2	High mobility group box 2	-1531, -1520, -1087
**Cell Cycle/Differentiation**			
[genebank:NM_006763]	BTG2	B-cell translocation gene 2 π	[76]
[genebank:NM_002166]	ID2	Inhibitor of DNA binding 2 π	-571*, -174
[genebank:X54941]	CKS1B	CDC28 protein kinase 1B	-1416, -1406* -717, -647, -637*, -646 -636*
[genebank:NM_004701]	CCNB2	Cyclin B2	-1387
**Growth**			
[genebank:NM_000598]	IGFBP3	Insulin-like growth-factor binding protein 3 π	-1729
**Miscellaneous**			
[genebank:NM_000674]	ADORA1	Adenosine A1 receptor	[25]
[genebank:NM_000201]	ICAM1	Intracellular adhesion molecule 1 π	[25]
[genebank:NM_002421]	MMP1	Matrix metalloproteinase 1	[25]
[genebank:X13895]	SAA3P	Serum amyloid A3 pseudogene	-1790*
[genebank:NM_002291]	LAMβ1	Laminin, β 1	-263, -253
[genebank:NM_004119]	FLT3	FMS related tyrosine kinase 3	-295, -285*
[genebank:NM_001365]	DLG4	Discs, large homolog 4	-1425, -1415*, -544*
[genebank:M22001]	F13A1	Coagulation factor X111, A1 polypeptide	-404, -72*

LPS responsive genes that have been shown to be NF-κB targets (Reference is indicated) or contain putative κB sites. The position of the site in relation to the initiator ATG is indicated and * indicates the site is found on the reverse strand. π indicates genes validated using real time PCR.

**Table 2:** Genes responsive to LPS that do not contain putative κB in their upstream sequences

Accession No	Symbol	Gene Name
**Chemokines and receptors**		
[genebank:NM_021006]	CCL3L1	Chemokine, CC motif, ligand 3-like protein 1 π #
[genebank:NM_002988]	CCL18	Chemokine, CC motif, ligand 18
[genebank:NM_005623]	CCL8	Chemokine, CC motif, ligand 8 π #
[genebank:NM_002991]	CCL24	Chemokine, CC motif, ligand 24 #
[genebank:NM_001838]	CCR7	Chemokine, CC motif, receptor 7 #
[genebank:NM_002620]	PF4V1	Platlet Factor 4, variant 1
**Angiogenesis**		
[genebank:NM_003246]	THBS1	Thrombospondin 1 #
[genebank:NM_012484]	HMMR	Hyaluronan-mediated motility receptor #
**Cytokines and Receptors**		
[genebank:NM_003853]	IL18 RAP	Interleukin 18 receptor accessory protein #
[genebank:NM_000565]	IL6R	Interleukin 6 receptor #
**Apoptosis**		
[genebank:U75285]	AIP4	Apoptosis inhibitor 4 (survivin)
[genebank:NM_021127]	PMAIP1	Phorbol 12 myristate 13 acetate induced protein 1 *#
[genebank:NM_014456]	PDC4	Programmed cell death 4 π
[genebank:X75252]	PBP	Prostatic binding protein #
**Transcription**		
[genebank:NM_001211]	BUB1β	Budding uninhibited by benzimidazoles 1, S. Cerevisiae homolog of B π#
[genebank:X52317]	H2FAZ	H2A histone family member Z
[genebank:NM_002128]	HMGB1	High mobility group box 1
[genebank:NM_006618]	JARID1B	Jumonji, AT-rich interactive domain 1B π #
[genebank:NM_006347]	PPIH	Peptidyl-prolyl isomerase H #
[genebank:NM_005030]	PLK1	Polo-like kinase #
[genebank:NM_001067]	TOP2a	Topoisomerase, DNA, 11, α π
**Growth**		
[genebank:NM_000222]	KIT	v-kit Hardy-Zuckerman 4 feline sarcoma viral oncogene homolog
**Miscellaneous**		
[genebank:NM_019111]	HLADRα	Major histocompatibilty complex, class II, DR α π
[genebank:D14657]		K1AA0247 #
[genebank:NM_006062]	SMYD5	SMYD family member 5 π

π Indicates those genes validated using real time PCR. # Indicates those genes that contain putative IRF-3 sites [see Additional file 1 for position].

**Table 3:** Comparison of microarray and real time PCR data

	Microarray		Taqman	
		
Gene	1 hr LPS	4 hr LPS	1 hr LPS	4 hr LPS
**Chemokines and receptors**				
CCL3	5.744	2.200	1622 (± 322)	141 (± 27)
CCL3L1	5.708	2.603	56 (± 3)	4 (± 1)
CCL4	4.769	3.399	7047 (± 836)	2481 (± 714)
CCL8 *	1.859	NC	8 (± 2)	94 (± 38)
CCL22	NC	4.021	NC	135 (± 28)
CXCL1 *	1.272	NC	258 (± 41)	250 (± 21)
CXCL2	3.938	2.787	243 (± 85)	68 (± 13)
CXCL8 *	NC	1.957	1016 (± 57)	491 (± 56)
CXCR4	NC	-1.493	NC	-4 (± 1)
**Cytokines and receptors**				
IL-1β	4.649	4.289	3490 (± 254)	1891 (± 135)
IL-1RN *	NC	1.830	4	6 (± 2)
PBEF	1.008	3.029	5 (± 2)	19 (± 1)
TNF-α	4.808	2.116	392 (± 55)	39 (± 4)
**Stress**				
COX-2	3.673	1.333	166 (± 34)	29 (± 8)
SOD2	4.433	5.620	44 (± 7)	264 (± 37)
TXNIP	-3.757	-1.638	-11 (± 2)	-2 (± 1)
**Apoptosis**				
cIAP2 *	NC	1.151	19 (± 3)	112 (± 11)
GADD45β	1.212	1.323	7 (± 1)	8 (± 1)
PDCD4	NC	-1.048	NC	-7 (± 1)
PMAIP1	2.604	NC	7 (± 1)	NC
TNFAIP3	2.905	2.526	104 (± 17)	44 (± 8)
**Transcription**				
C-MYB	NC	-1.584	NC	-5 (± 1)
TOP2α	NC	-1.561	NC	-3
C-REL *	NC	2.869	4 (± 1)	38 (± 3)
IκB-α	4.024	3.006	40 (± 4)	19 (± 2)
NFκB1	1.150	3.427	4 (± 1)	36 (± 2)
**Cell cycle and Differentiation**				
BTG2 *	2.023	NC	116 (± 21)	32 (± 7)
BUB1β	NC	-1.244	NC	-3
ID2	NC	1.299	NC	4 (± 1)
JARID1B π	2.675	4.061	NC	NC
**Growth**				
IGFBP3 *	NC	4.182	43 (± 9)	1420 (± 167)
**Adhesion**				
ICAM1 *	NC	1.188	6	70 (± 44)
**Miscellaneous**				
HLA-DRα π	NC	1.593	NC	NC
SMYD5 π	3.699	2.417	NC	NC

Real time PCR results correspond to mean fold change normalised to GAPDH from 3 biological replicates and SEM is indicated. Genes where significantly different results between microarray and Taqman data was observed are indicated with an π. Genes which were originally scored as no change (NC) based on microarray data (at one time point) but where the increased sensitivity of Taqman revealed a change are indicated with an *. Note, any gene that showed a mean fold change between 2 and -2 was scored as NC in Taqman.

**Table 4:** Comparison of LPS response in U937 cells, THP-1 and PBMC macrophages.

					PBMC macrophage			
					
	U937		THP-1		Donor A		Donor B	
	
	Time of LPS treatment							
Gene	1	4	1	4	1	4	1	4

**Chemokines/Receptors**								
CCL3	18	19 (± 3)	1622 (± 322)	141 (± 27)	22	57	90	89
CCL3L1	ND	21 (± 7)	56 (± 3)	4 (± 1)	62	51	271	83
CCL4	30 (± 11)	228 (± 63)	7047 (± 836)	2481 (± 714)	31	154	197	445
CCL8	ND	ND	8 (± 2)	94 (± 38)	11	134	42	175
CCL22	NC	NC	NC	135 (± 28)	NC	3	NC	NC
CXCL1	ND	ND	258 (± 41)	250 (± 21)	74	86	308	91
CXCL2 *	15 (± 3)	8 (± 3)	243 (± 85)	68 (± 13)	97	35	260	16
CXCL8	7 (± 1)	9 (± 3)	1016 (± 57)	491 (± 56)	118	280	282	178
CXCR4	NC	NC	NC	-4 (± 1)	2	NC	2	-4
**Cytokines/Receptors**								
IL-1β	5	6 (± 1)	3490 (± 254)	1891 (± 135)	139	277	905	325
IL-1RN	NC	NC	4	6 (± 2)	11	56	21	75
PBEF	NC	2	5 (± 2)	19 (± 1)	2	24	2	37
TNF-α*	9 (± 2)	3	392 (± 55)	39 (± 4)	164	144	626	233
**Stress**								
COX-2	14 (± 4)	17 (± 5)	166 (± 34)	29 (± 8)	291	888	432	333
SOD2 *	2	7	44 (± 7)	264 (± 37)	6	42	7	31
TXNIP	NC	NC	-11 (± 2)	-2 (± 1)	-3	-2	-3	-3
**Apoptosis**								
cIAP2 *	6 (± 1)	12	19 (± 3)	112 (± 11)	11	54	16	26
GADD45β	NC	NC	7 (± 1)	8 (± 1)	4	18	10	24
PDCD4	NC	NC	NC	-7 (± 1)	NC	-2	NC	-3
PMAIP1	NC	NC	7 (± 1)	NC	127	74	213	34
TNFAIP3 *	17 (± 1)	15 (± 3)	104 (± 17)	44 (± 8)	54	63	83	39
**Transcription**								
C-MYB	NC	NC	NC	-5 (± 1)	NC	NC	-2	3
TOP2α	NC	NC	NC	-3	NC	-3	NC	-2
C-REL	NC	NC	4 (± 1)	38 (± 3)	2	15	3	8
IκB-α*	8 (± 2)	6	40 (± 4)	19 (± 2)	18	17	20	16
NFκB1	NC	2	4 (± 1)	36 (± 2)	3	35	4	27
**Cell cycle/Differentiation**								
BTG2	4 (± 1)	5 (± 2)	116 (± 21)	32 (± 7)	5	3	8	NC
BUB1β	NC	NC	NC	-3	NC	-5	NC	-4
ID2	NC	NC	NC	4 (± 1)	NC	2	NC	2
JARID1B	NC	NC	NC	NC	NC	2	NC	NC
**Growth**								
IGFBP3	ND	ND	43 (± 9)	1420 (± 167)	ND	12	ND	46
**Adhesion**								
ICAM1 *	2	5 (± 1)	6	70 (± 44)	7	19	10	20
**Miscellaneous**								
HLA-DRα	2 (± 4)	NC	NC	NC	NC	2	NC	2
SMYD5	NC	NC	NC	NC	NC	-2	NC	-2

Real time PCR results correspond to mean fold change normalised to GAPDH from 3 biological replicates of U937 cells treated with LPS and SEM is indicated. Genes showing an identical expression profile between THP-1 cells, U937 cells and PBMC derived macrophages from both donors are indicated with an *
